# Dynamic cell culture modulates colon cancer cell migration in a novel 3D cell culture system

**DOI:** 10.1038/s41598-024-69261-2

**Published:** 2024-08-14

**Authors:** M. Mohamadian Namaqi, F. Moll, S. Wiedemeier, A. Grodrian, K. Lemke

**Affiliations:** Department of Bioprocess Engineering, Institute for Bioprocessing and Analytical Measurement Techniques e.V. (iba), Heilbad Heiligenstadt, Germany

**Keywords:** Dynamic 3D cell culture, Colon cancer, Cancer cell migration, Biological techniques, Biotechnology, Cancer

## Abstract

The progression of cancer cell migration, invasion and subsequent metastasis is the main cause of mortality in cancer patients. Through creating more accurate cancer models, we can achieve more precise results, which will lead to a better understanding of the invasion process. This holds promise for more effective prevention and treatment strategies. Although numerous 2D and 3D cell culture systems have been developed, they poorly reflect the in vivo situation and many questions have remained unanswered. This work describes a novel dynamic 3D cell culture system aimed at advancing our comprehension of cancer cell migration. With the newly designed cultivation chamber, 3D tumor spheroids were cultivated within a collagen I matrix in the presence of fluid flow to study the migration of cancer cells from spheroids in the matrix. Using light sheet microscopy and histology, we demonstrated that the morphology of spheroids is influenced by dynamic culture and that, in contrast to static culture, spheroids in dynamic culture are characterized by the absence of a large necrotic core. Additionally, this influence extends to an increase in the size of migration area, coupled with an increase in expression of some genes related to epithelial-mesenchymal transition (EMT). The results here highlight the importance of dynamic culture in cancer research. Although the dynamic 3D cell culture system in this study was used to investigate migration of one cell type into a matrix, it has the potential to be further developed and used for more complex models consisting of different cell types or to analyze other steps of metastasis development such as transendothelial migration or extravasation.

## Introduction

Cancer is still one of the deadliest diseases worldwide, with colorectal cancer (CRC) ranking as the third most frequent cancer-related cause of death. In 2020 alone, CRC caused over 900,000 deaths globally, with 1.9 million new cases reported^1^. The dominant cause of this mortality is metastasis, which begins with epithelial-to-mesenchymal transition (EMT). EMT allows cancer cells to spread from the primary tumor into the blood circulation. This complex cellular process involves epithelial cells acquiring a mesenchymal phenotype that helps cells adapt to the dynamic tumor microenvironment and metastasize^2^. After metastatic tumor cells reach distant organs, they undergo the reverse transition into metastatic lesions through mesenchymal-to-epithelial transition (MET)^3^. Evidence shows that there are multiple transitional states, and EMT progresses gradually to a partial EMT (pEMT) state, characterized by incomplete loss of epithelial markers and partial gain of mesenchymal markers^4,5^. Despite extensive research efforts, a lot of mechanisms underlying tumor development and progression are still poorly understood due to their complex nature. To address this, many attempts have been undertaken to investigate the effect of genetic, environmental and mechanical factors on cancer^6,7^.

While conventional 2D cell culture techniques have been widely employed and are cost-effective, their limitations in accurately modeling complex processes have been increasingly recognized. Growing cells as a monolayer on a flat surface fails to replicate the intricate microenvironment of tumors, often leading to disparities between research outcomes and clinical observations^8^. Recognizing the significant role of the microenvironment in modulating cell behavior, researchers have developed different three-dimensional (3D) cell culture models. These models better recapitulate the different phases of cancer due to their capacity to demonstrate tissue structure and function, as well as in vivo cell morphology and cell–cell interactions. Among these models, spheroids—3D aggregates of cells—have emerged as promising models for studying tumor dynamics, capturing essential features such as cell–cell interactions and gradients of signaling factors, nutrients, and oxygen^9,10^.

However, in order to establish more accurate models, additional factors must be considered. The presence of extracellular matrix (ECM) in cancer studies is crucial to obtain a deeper understanding of cancer mechanisms. Matrix not only maintains cell homeostasis^11^, but also plays an important role in mechanical and chemical communication between cells^12^, influencing various cell behaviors such as cell proliferation and survival^11^. Additionally, ECM adhesion facilitates cell movement, which is a crucial step in cancer cell migration^13^, stressing the need to incorporate ECM in in vitro cancer models.

Nonetheless, limitations in oxygen and nutrient delivery within 3D cell culture systems with matrix have been described, primarily due to the increased distance to the culture medium. While similar challenges exist in vivo, blood perfusion and angiogenesis facilitate oxygen and nutrient delivery^14,15^. In order to mimic this, integrating fluid flow into cell culture systems has been proposed to improve oxygen and nutrient diffusion while also promoting waste removal. In a study using 2D cell cultures and traditional cell culture plastic ware, it has been shown that dynamic systems maintain a more consistent environment in comparison to medium exchanges in static systems, resulting in reduced expression of DNA damage-inducible transcript 3 (DDIT3), a hallmark of cellular stress^14,16^. Furthermore, dynamic culture has been shown to alter cell behavior, for instance promoting the proliferation of human oral keratinocytes cultured in collagen and chondroitin matrices^17^ or inhibition of chondrogenesis in single bovine articular chondrocytes in a similar system in which collagen sponges were suspended within a column under dynamic culture conditions^18^. In cancer research, micro- and macrofluidic systems have also been developed to meet a range of requirements. For instance, a 3D culturing system was developed for the cultivation of cancer spheroids and drug screening in an expedited fashion^19^. Although it accelerates the therapeutic testing process by employing convective nutrient supply, the absence of matrix prevents accurate simulation of solid tumors. Other research focuses on mimicking specific physiological conditions, such as engineered microvessels on a metastasis chip that can recreate angiogenesis and intravasation of dispensed cells in a matrix. The system was purposefully designed to explore cancer-endothelial cell interactions, allowing for the quantification of transendothelial migration^20^. In a similar study, a Plug-and-Play device aimed at studying the metastatic cascade revealed that cancer cells, in monolayer culture or suspended in a matrix, tend to proliferate and migrate under dynamic culture condition^21^. In addition, some research has focused on the role of fluid flow in EMT. Cultivation of non-small cell lung cancer within a multi-channel microfluidic model platform showed that, compared to traditional static cultures, flow triggers changes in cellular morphology and EMT^22^. However, further research is required to consider tumors as an initial stage for cancer cell migration and metastasis.

Despite advancements in fluidic systems, there remains a shortage of in vitro models supporting the cultivation of spheroids within an matrix under dynamic conditions. Addressing this gap, we have designed and developed a novel in vivo-like system. Our system features a cultivation chamber consisting of a well filled with collagen I matrix and spheroids, with nutrients delivered to the spheroids from the circulating medium in the channel above the well. Through our investigations, we observed significant differences in cancer cell migration under dynamic compared to static conditions, demonstrating the potential of such a system for precise cancer studies. Discoveries facilitated by these models, will contribute to a better understanding of the underlying mechanism of cancer cell migration and metastasis.

## Results

### Cultivation of HCT-116-GFP spheroids in a collagen I matrix supports cell migration and alters gene expression

**Figure 1:**
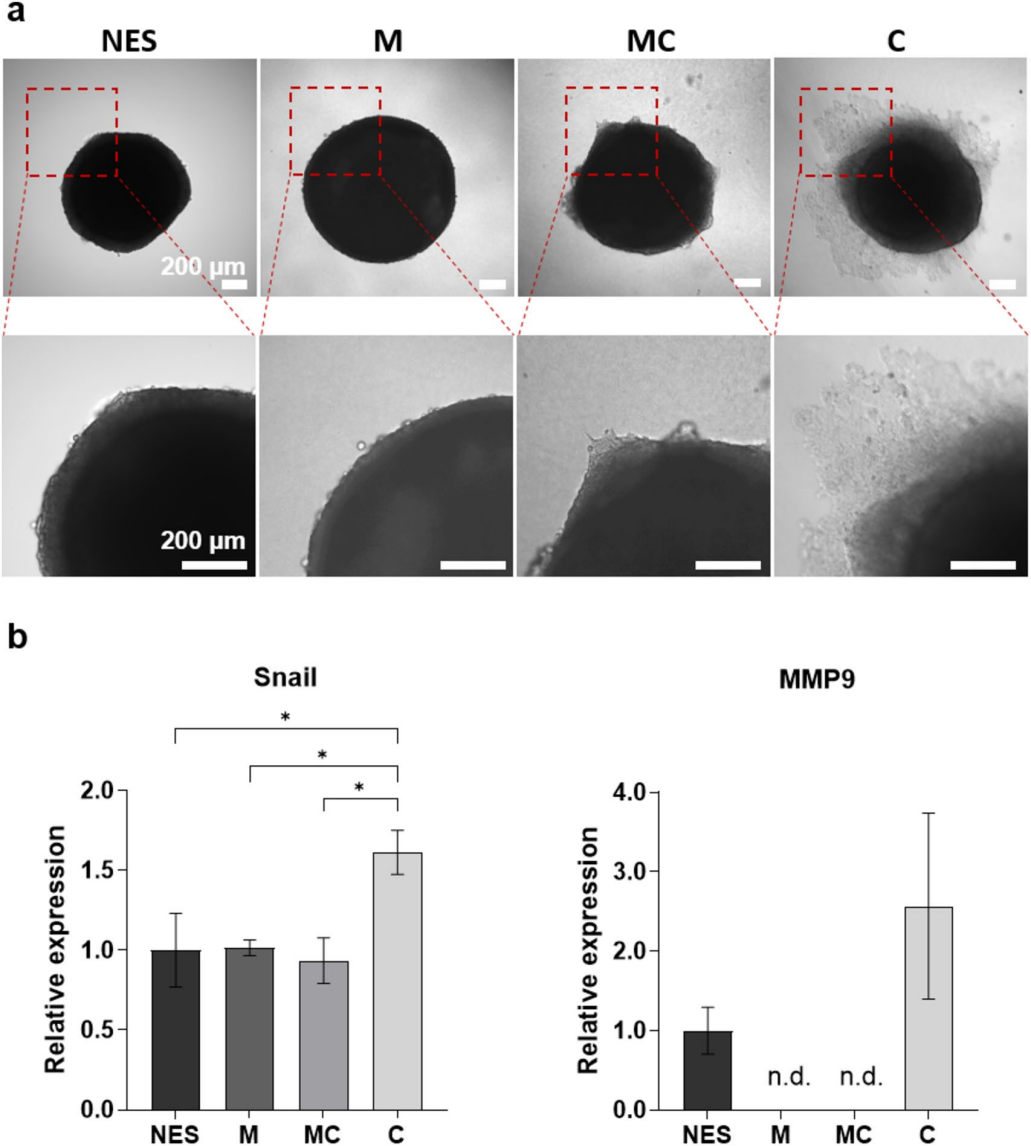
Effect of different matrices on cell migration in HCT-116-GFP spheroids cultured for seven days. (**a**) Representative images of non-embedded and embedded HCT-116-GFP spheroids in different matrices. Upper panel shows the whole spheroid; lower panel shows zoomed-in section. Scale bar: 200 µm. (**b**) Analysis of mRNA expression of Snail and MMP9 in non-embedded and embedded HCT-116-GFP spheroids in different matrices. NES: non-embedded spheroids, M: Matrigel^®^, C: collagen I and MC: mixture of Matrigel^®^ and collagen I. n = 3. n.d. = not detected (ct-value > 35.00). Data are shown as mean ± SD (*p < 0.05).

In the initial investigation, different extracellular matrix substrates were evaluated to identify an appropriate matrix for studying cancer cell migration. The effects of Matrigel^®^, collagen I and a mixture of both on migration were compared using HCT-116-GFP spheroids. Matrigel^®^, derived from the Engelbreth-Holm-Swarm (EHS) mouse sarcoma, is rich in different proteins such as laminin and collagen IV, compared to the fibrillar collagen I matrix. Both matrices are commonly used in cancer migration and invasion assays. HCT-116-GFP spheroids which had formed over four days were embedded in either a Matrigel^®^ (M), collagen I (C) or Matrigel-collagen l (MC) matrix. The embedded spheroids were cultured for seven days and subsequently imaged using microscopy. Cell migration was defined as cells moving away from the spheroid. The images revealed migration of HCT-116-GFP cells into collagen l, while migration was not observed in pure Matrigel^®^. Here embedded spheroids were fully spherical and only increased in size over the course of cultivation (Supplementary Fig. S1). Spheroids cultured in the Matrigel^®^-collagen l matrix also did not show classical migration of cancer cells but in contrast to pure Matrigel^®^ the spheroids seemed to be less spherical and formed tip-like structures protruding into the matrix as a result of the added collagen I (Fig. 1a).

Further investigation focused on the expression of genes associated with cell migration and EMT: Snail and matrix metalloproteinases (MMP) 9 (Fig. 1b). In the initial stages of EMT, several signaling pathways cooperate to activate Snail expression, leading to the increased expression of other genes such as MMPs^23^. Analysis revealed a 1.6-fold increased Snail expression within spheroids embedded in collagen l compared to spheroids cultured non-embedded in liquid overlay (NES) for the same time. No such upregulation was observed in spheroids cultured in Matrigel^®^ and Matrigel^®^-collagen l matrix. Expression of MMP9 could not be detected in spheroids cultured in either Matrigel^®^ or Matrigel^®^-collagen l matrix, indicating a reduction in gene expression compared to non-embedded spheroids. Although not significant, in spheroids cultured in collagen I MMP9 expression was increased compared to non-embedded spheroids. Consequently, collagen I was selected as the matrix for subsequent experiments to investigate the effect of dynamic culture on spheroid migration.

### Development of the polycarbonate-based cultivation chamber for a dynamic 3D cell culture system

**Figure 2:**
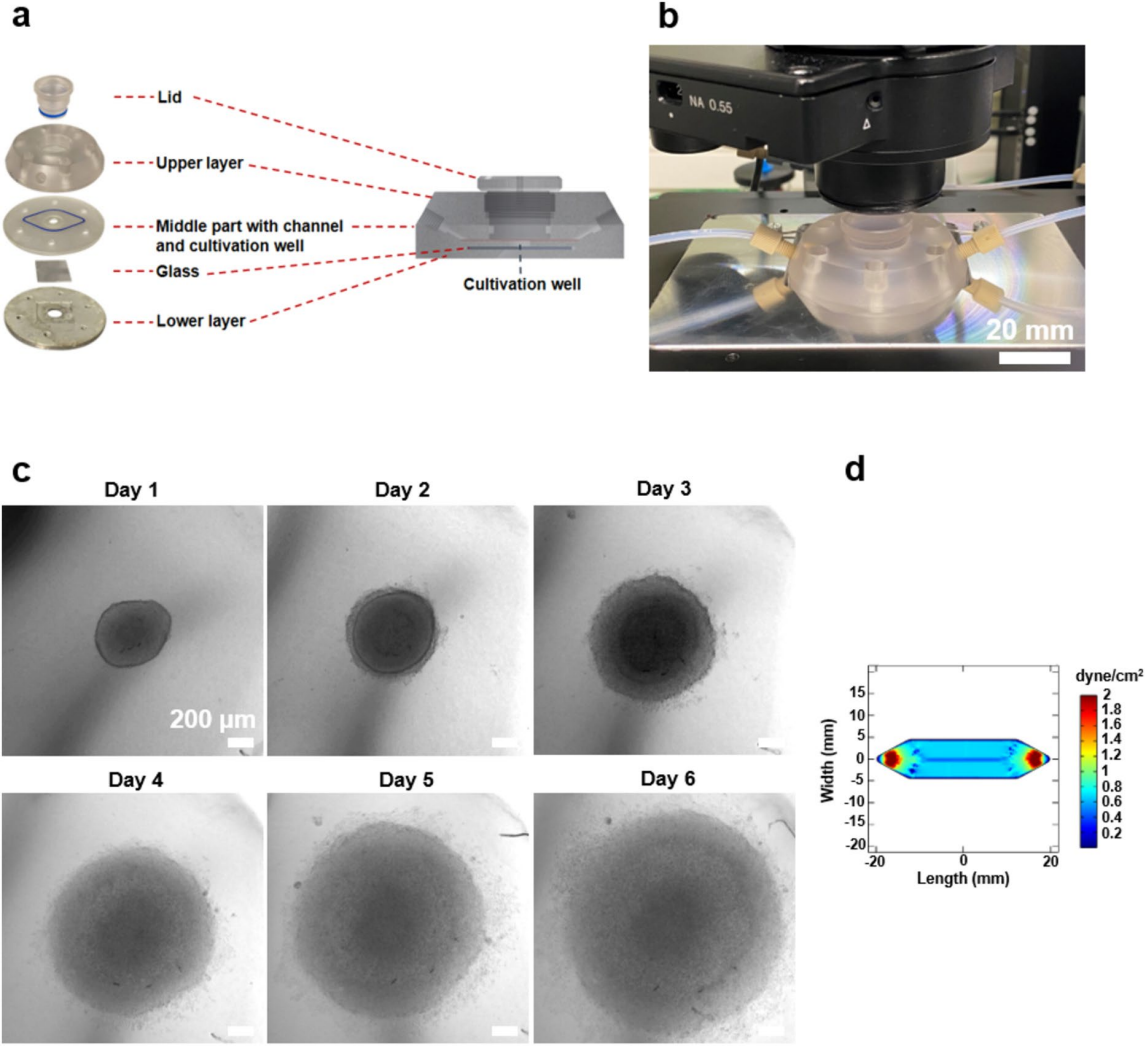
Cultivation chamber description and simulation of shear stress. (**a**) Schematic representation of the cultivation chamber and its layers: The cultivation chamber is made of different layers, a well for loading collagen gel and spheroids, a channel that allows medium to flow over the well. (**b**) Picture of the developed microscope stage holder (silver) with the cultivation chamber and connected tubing. Scale bar: 20 mm. (**c**) Online images showing HCT-116-GFP spheroids embedded in collagen I and cultured within the chamber under dynamic conditions for 6 days. Scale bar: 200 µm (**d**) Simulation of shear stress in the channel and over the well by COMSOL at flow rate of 1 ml/min.

A new in vivo-like system was developed to investigate cancer cell migration in the collagen I matrix. The cultivation chamber was fabricated using layers of polycarbonate, glass and aluminum (Fig. 2a). While poly(dimethylsiloxane) (PDMS) is commonly used in micro- and macrofluidic systems, polycarbonate was chosen for the upper and middle layers due to its superior adsorption properties and biocompatibility compared to PDMS^24^. The upper layer contains the lid and connectors for tubing, while the middle layer contains a channel for circulating medium and a well for spheroid cultivation in the collagen I matrix. To study cell migration, it is essential to provide sufficient space for cellular movement. Despite the capability of our system to culture single cells in collagen gel under dynamic conditions, the study focused on spheroids as a tumor model. To ensure uniform size on the day of culturing under static and dynamic conditions, spheroids were first generated in 96-well plates. After 4 days, when they were well-formed and sizable, 350.89 ± 12.2 diameter spheroids (Supplementary Fig. S2) were transferred into the system. The well, with dimensions of 7 mm in diameter and 2 mm in height, allows for the cultivation of two spheroids. The bottom of the well is composed of glass, which allows for online visualization of embedded cells throughout the culturing period. A new microscope stage holder was designed to ensure precise positioning of the cultivation chamber during imaging (Fig. 2b and c). In addition, the glass bottom was coated with poly(2-hydroxyethyl methacrylate) (pHEMA) to prevent cell adhesion. Spheroids obtain nutrients from the circulating medium in the channel above the well, facilitated by a radial pump system designed to provide continuous flow. The flow rate of 1 ml/min causes shear stress of 0.8 dyne/cm^2^, mirroring the physiological shear conditions observed in venous circulation (0.5–4 dyne/cm^2^)^25^ (Fig. 2d). The system is designed for multiple uses, and due to the temperature stability of the utilized materials, it can be sterilized through autoclaving.

In conclusion, the newly developed polycarbonate-based cultivation chamber provides an in vivo-like environment for studying cancer cell migration in the collagen I matrix, offering precise control over dynamic conditions and enabling real-time visualization of cellular dynamics, setting the stage for subsequent experimentation on invasion dynamics.

### Dynamic culture significantly stimulates the migration of colon cancer cells in collagen I matrix

**Figure 3:**
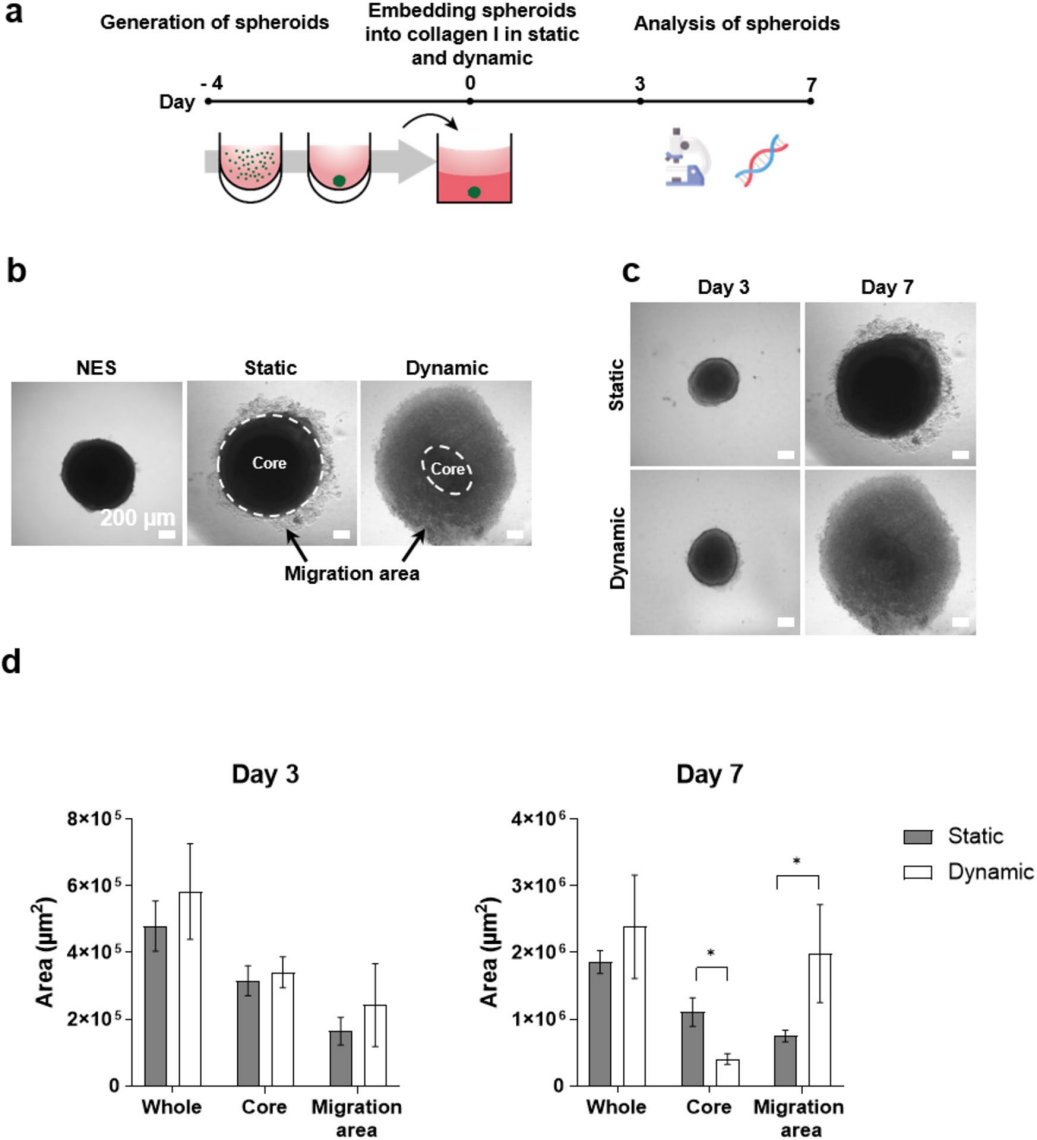
Investigation of migration for HCT-116-GFP in collagen I matrix in the dynamic 3D cell culture system. (**a**) Experimental workflow of spheroid generation by liquid overlay technique, embedding in collagen I matrix and analysis through microscopy and gene expression. (**b**) Representative images of spheroids cultured in liquid overlay (non-embedded (NES)), static or dynamic conditions illustrating the distinct core (dotted outline) and migration areas used for analysis. (**c**) Representative images of embedded spheroids in static and dynamic culture conditions. Quantification of spheroid migration on day 3 and day 7 (**d**), n = 8. Scale bar: 200 µm. Data are shown as mean ± SD (*p < 0.05).

To investigate the effect of dynamic culture on migration of colon cancer cells, two 4-day-old HCT-116-GFP spheroids were embedded in collagen I (1.8 mg/ml) inside the cultivation chamber well and a static control well (Fig. 3a). The migration area was analyzed using bright field microscopy images on day 3 and 7 of cultivation. This revealed similarity on day 3, but a noticeable difference in their appearance on day 7 (Fig. 3c). Cells from the embedded spheroids start migrating in the collagen matrix, resulting in the identification of two distinct areas: the core and migration area (Fig. 3b). The migration area was calculated by subtracting the core from the whole area of the spheroid, indicating how far the cancer cells move and invade into the matrix. Analysis revealed a significant difference between static and dynamic conditions on day 7, with spheroids exhibiting a smaller (~ 0.4 fold) core area and a larger (~ 2.5 fold) migration area under dynamic condition, despite no significant change in the whole area (Fig. 3d). The increased migration area observed under dynamic conditions emphasizes the pivotal role that dynamic culture plays in promoting the movement of HCT-116-GFP cells.

### Dynamic culture affects 3D morphology of embedded spheroids

**Figure 4:**
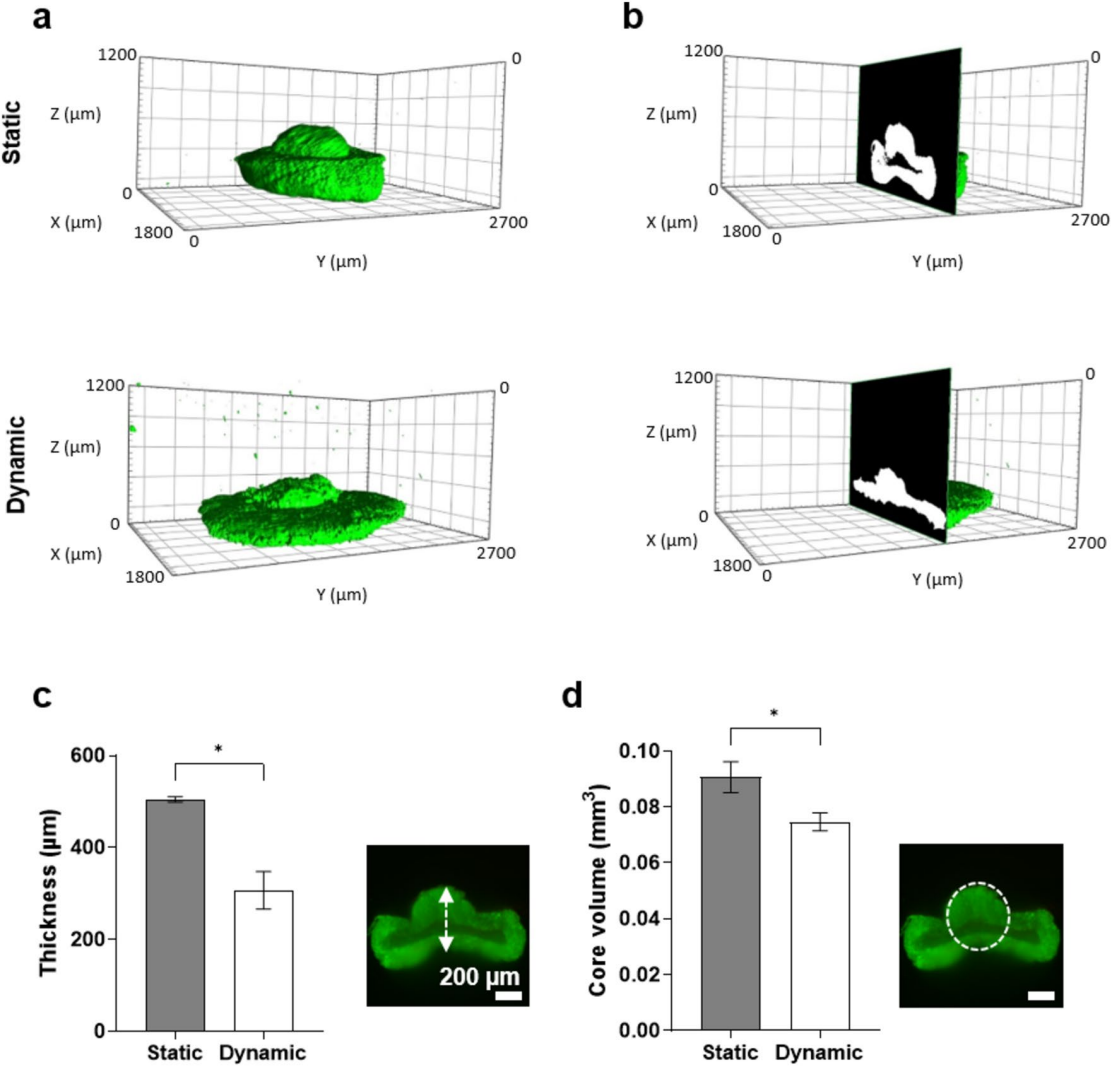
Light sheet fluorescence microscopy images of embedded HCT-116-GFP spheroids. (**a**,**b**) The morphology and internal fluorescence intensity of HCT-116-GFP spheroids in static and dynamic conditions. The thickness (**c**) and the core volume (**d**) of spheroids in static and dynamic conditions. Scale bar: 200 µm. Data are shown as mean ± SD. n = 3 (*p =  < 0.05).

Spheroid morphology is recognized as an important factor in experimental standardization^26^. Considering the limitations of 2D imaging, which provides only restricted information and lacks depth required for comprehensive analysis, embedded spheroids were visualized using light sheet fluorescence microscopy. The utilization of 3D imaging techniques allows for a more in-depth examination of spheroid morphology and internal structure. The images revealed significant variations in spheroid morphology under different conditions (Fig. 4a). When analyzing the middle layer of the spheroids cultured in static condition, an area without GFP-fluorescence signal was observed, indicating a lack of cells in this area therefore suggesting the presence of a necrotic core in these samples, whereas this was not observed in spheroids cultured in the cultivation chamber (Fig. 4b). The morphology was analyzed by quantifying core volume and thickness of the core area. Spheroids cultured in the cultivation chamber were flatter than spheroids in static culture, which were more than 1.6- fold thicker (Fig. 4c). This also leads to a 1.2-fold increase of the core volume of static samples (Fig. 4d). These data demonstrate that dynamic culture has a discernible impact on both spheroid morphology and possibly the development of a necrotic core.

### Spheroids show enhanced proliferation and reduced apoptosis under dynamic culture conditions

**Figure 5:**
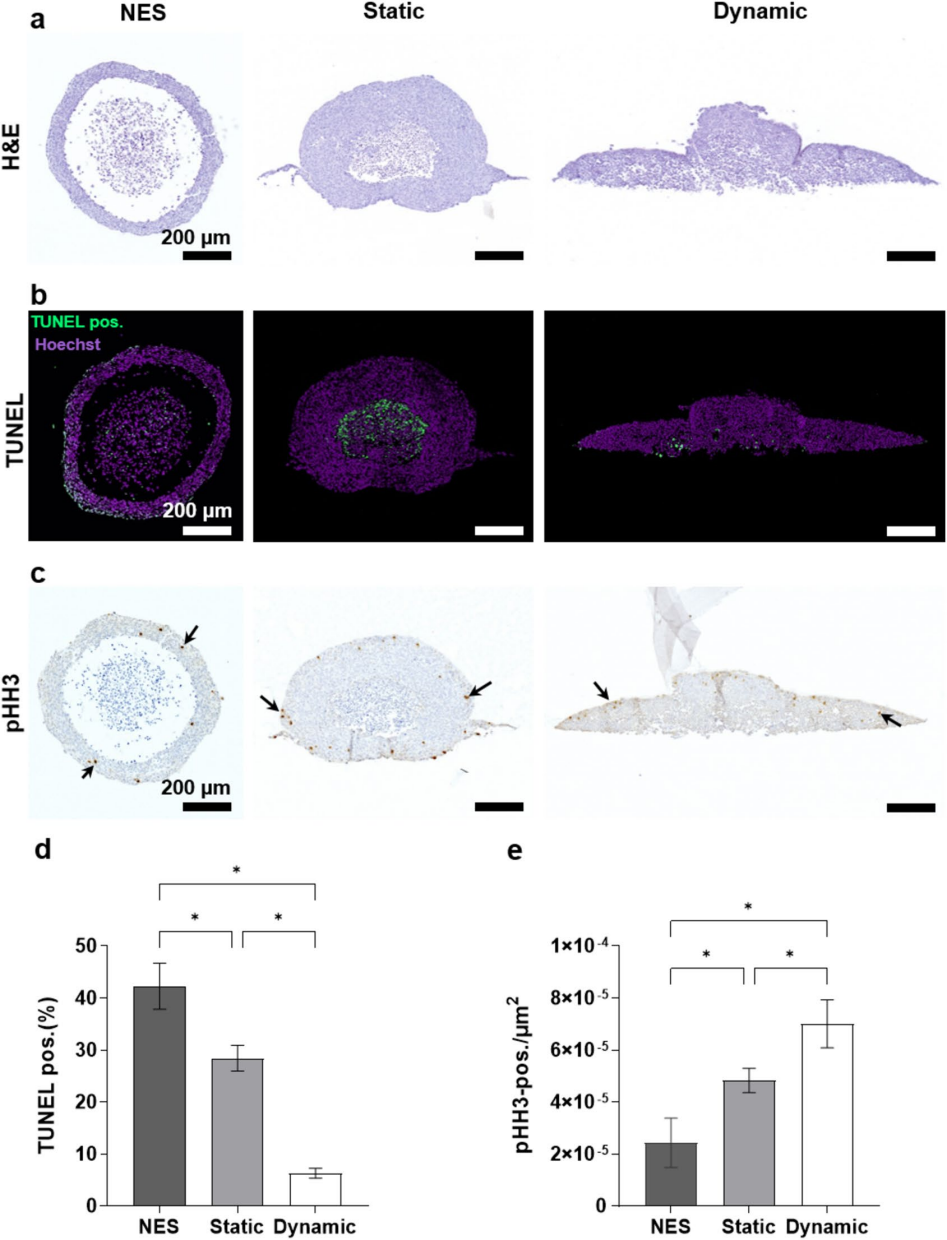
Histology staining of HCT-116-GFP spheroids in non-embedded spheroids (NES), static and dynamic culture conditions. (**a**) H&E staining. (**b**) TUNEL assay. (**c**) Immunohistochemistry with specific antibody against pHH3, arrows indicate pHH3-positive cells. (**d**) Quantitative result of TUNEL assay, the number of TUNEL-positive cells (green) is normalized to the total nuclei (purple). (**e**) Quantitative result of pHH3 staining, the number of pHH3-positive cells is normalized to the total area of spheroids. Scale bar: 200 µm. Data are shown as mean ± SD. n = 4 (*p =  < 0.05).

The observed variations in morphology and internal structure of spheroids in 3D images under static and dynamic culture conditions prompted a more detailed exploration of these samples using histological methods. The distinct morphologies under different conditions were confirmed through H&E staining. Using LSFM analysis a necrotic core can only be assumed through the lack of fluorescence signal in the center of spheroids; however, using histological staining, dead cell debris can also be detected. Specifically, a significant necrotic core was observed in non-embedded spheroids and spheroids in static condition, whereas no necrotic core was found in embedded spheroids under dynamic condition (Fig. 5a). Generally non-embedded spheroids showed only a thin layer of healthy cells and a large area completely lacking cells. This was not observed in embedded spheroids where the healthy cell layer was also much thicker.

For further analysis of cell death, the presence of DNA fragmentation was evaluated using the TUNEL (terminal deoxynucleotidyl transferase nick-end labeling) assay, which labels DNA strand breaks^27^. Here TUNEL staining of sectioned spheroids was used to determine the influence of dynamic culture on the number of apoptotic cells (Fig. 5b). Apoptosis was found to be 6.7-fold lower under dynamic condition compared to non-embedded spheroids, and 4.5-fold lower compared to static condition (Fig. 5d). For embedded static spheroids, staining was mainly localized within the necrotic core, where the non-embedded spheroids also showed apoptotic cells within the outer layer of the spheroid. In spheroids cultured under dynamic condition staining was only observed on the bottom of the spheroid, meaning the area furthest away from oxygen and nutrient supply.

Monitoring phospho-Histone H3 (pHH3) expression allows for the identification of cells undergoing mitosis^28^. Through pHH3 staining, it was demonstrated that proliferative cells were distributed on the outer layer of spheroids regardless of the culture conditions (Fig. 5c). Additionally, under dynamic culture condition, there was an approximately 1.5-fold higher number of pHH3-positive cells compared to static condition, while an approximately 2.9-fold higher number was observed compared to non-embedded spheroids (Fig. 5e).

Collectively, the findings show that the presence of fluid flow leads to increased cell proliferation and reduced apoptosis in HCT-116-GFP spheroids.

### Collagen I matrix and dynamic culture condition stimulate expression of EMT-related genes Snail and MMP9

**Figure 6:**
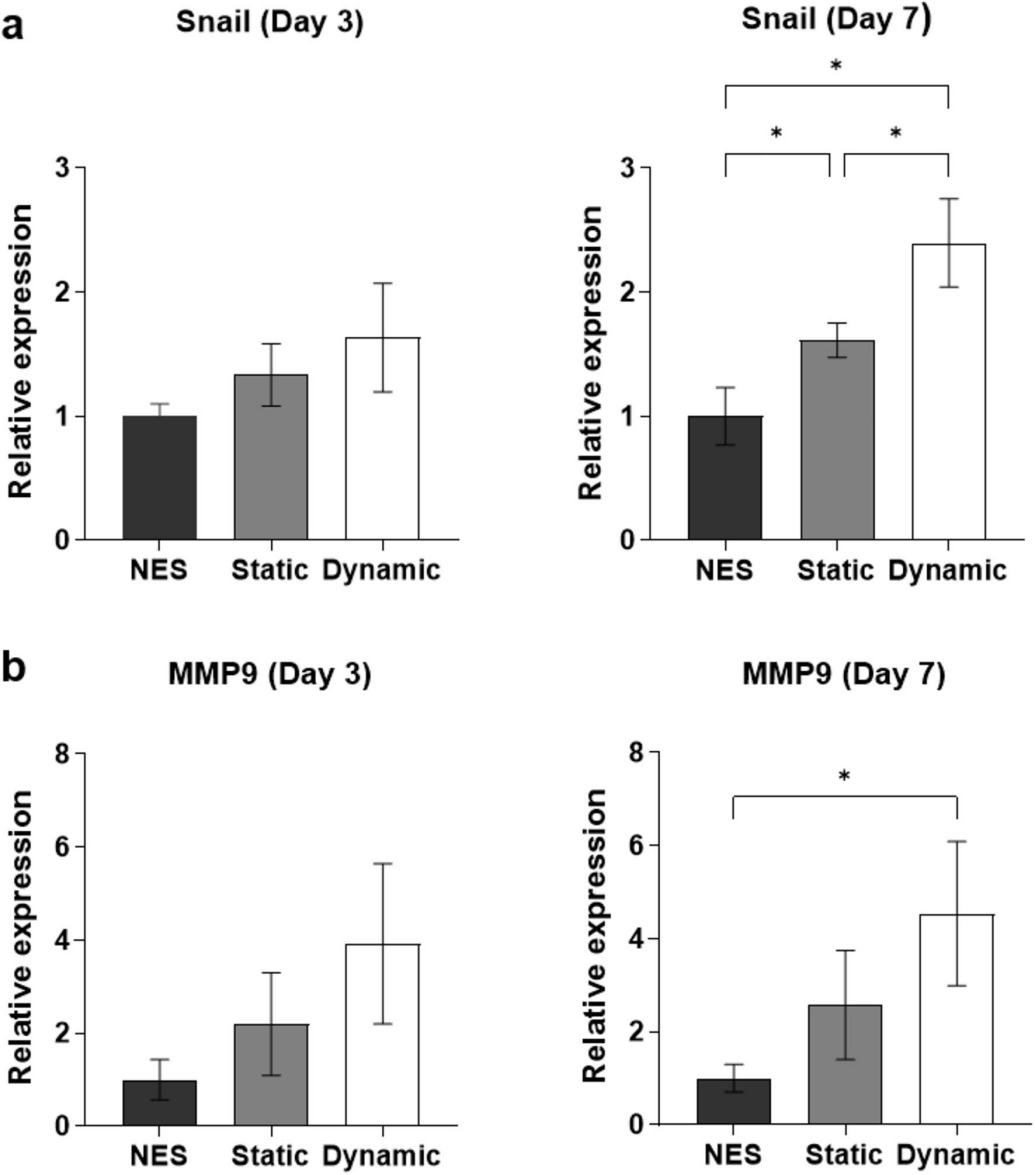
Gene expression analysis of cells in static and dynamic culture conditions. (**a**) Expression levels of Snail on day 3 and day 7. (**b**) Expression levels of MMP9 on day 3 and day 7. Gene expression was analyzed by qPCR using HSP90AB as reference gene (2^−ΔΔCT^ method). Data are shown as mean ± SD. n = 3 (*p =  < 0.05).

The increased migration area in dynamic culture condition, hints towards an activation of EMT processes. To verify this, the expression of EMT related genes was analyzed. As shown above, embedding HCT-116-GFP spheroids in the collagen I matrix leads to increased expression of both Snail and MMP9. Since the expression of these genes can be influenced by different microenvironmental stimuli ^29,30^ and is used as a marker for EMT, the effect of dynamic culture on the spheroids was analyzed on day 3 and 7 (Fig. 6). On day 3, the relative expression of both Snail and MMP9 is increased under dynamic culture condition but not significantly. The effect is more prominent on day 7, where the expression of Snail in static and dynamic culture conditions compared to non-embedded spheroids (NES) is significantly increased by 1.6 and 2.4 fold, respectively. MMP9 expression is 4.5 fold increased in dynamic culture condition compared to NES, but there is no significant difference to spheroids cultured under static culture condition. Despite an increase in the expression of Snail an MMP9 on day 7, no changes were observed in the expression of other mesenchymal markers such as Slug and Vimentin, and the expression of N-cadherin was significantly low in static and dynamic conditions compared to non-embedded spheroids. The expression of Integrin beta-4 and Catenin beta-1, which are shown to be involved in EMT^31,32^, remains unchanged under static and dynamic conditions (Supplementary Fig. S3).

Taken together, these findings show that dynamic culture is associated with changes in cancer cell migration behavior and its effects can initiate epithelial to mesenchymal transition in HCT-116-GFP spheroids.

## Discussion

In response to the increasing significance of in vivo-like models for gaining a comprehensive insight into biological processes and ensuring more reliable results, our study introduces a novel cultivation chamber designed for a dynamic 3D cell culture system. This system has been optimized to facilitate the monitoring of cancer migration under conditions that closely mimic the in vivo environment, including the presence of the extracellular matrix and fluid flow dynamics.

In recent years, various micro- and macrofluidic systems have emerged as valuable tools to study biological processes such as metastasis, differentiation of stem cells and drug screening^33^. These systems offer increased experimental capabilities and lead towards further automation. These innovative approaches provide the means to design and conduct experiments that more accurately reflect biological processes compared to traditional 2D cell monolayer experiments. Additionally, these advancements are the basis to reduce the need for laboratory animals in future studies. Given the significant differences between rodents and humans, despite their biological similarities, such a shift could mitigate costs and the possibility of clinical trial failures in addition to the ethical conflicts when using animals^34^.

Each of these systems has been specially designed and optimized to fulfill a distinct purpose. Various types of ECM, such as Matrigel^®^ and different collagens, have been used in the cultivation of different cell lines within microfluidic devices and dynamic culture systems. Although Matrigel^®^ has been used in migration and invasion research, some studies propose collagen I as a superior scaffold due to biophysical cues such as large pore size and fibrillar matrix architecture, which facilitates 3D migration and invasion^35^. Furthermore, collagen I triggers integrin linked kinase (ILK)-dependent pathways and induces EMT and cell migration^36^. In this study, we have confirmed that collagen I is a more appropriate matrix choice over Matrigel® for investigating cell migration in HCT-116 cells. Additionally, despite the widespread use of single cells within a matrix in many systems^37,38^, our strategy involves utilizing cancer spheroids as a tumor model. Spheroids offer advantages in cancer research, such as increased cell–cell and cell-ECM interactions and mimicking oxygen and nutrient gradients of a solid tumor. Besides the oxygen and nutrient gradients, in the body, vessels facilitate delivery of oxygen and nutrients to tumors. Considering this aspect is essential in 3D culture models, as limitations in oxygen and nutrient delivery arise due to the extended distance to the culture medium^15^. To address this, we have designed a channel above the cultivation chamber to realize the physiological supply of oxygen and nutrients to the tumor spheroid. Additionally, fluid flow in the body generates shear stress along the walls of blood vessels. At a flow rate of 1 ml/min, the shear stress exerted over the cultivation chamber well in our system is approximately 0.8 dyne/cm^2^, resembling the shear conditions found in venous circulation (ranging from 0.5 to 4 dyne/cm^2^)^25^. The newly designed chamber not only facilitates spheroid culture under dynamic culture condition but also offers the advantage of real-time observation and imaging, owing to transparency of the well.

The migration of tumor cells into the surrounding matrix is a significant aspect in cancer progression, as it represents one of the initial and critical steps towards metastasis^39^. Therefore, the quantitative assessment of cancer cell migration from a primary tumor offers valuable insights into invasion and metastasis. Conventional in vitro assays assessing tumor migration and invasion often use methods such as scratch wound healing and transwell migration in the absence of microenvironmetal factors such as matrix or fluid flow that have shown to influence the migration of cells in our system. Analyzing 2D images of embedded spheroids provides valuable insights into cell migration, with the migration area being a significant parameter commonly used in many studies^40^. The observation of a larger migration area in embedded spheroids cultivated within the cultivation chamber compared to static condition, stresses the importance of dynamic culture in modeling cancer migration. Additionally, the noticeable difference becomes more prominent after 7 days of cultivation compared to the third day, highlighting the need for dynamic culture systems facilitation long-term culture, consistent with findings of previous studies. For instance, changes in MDA-MB-231 cell morphology, results from the scratch wound healing assay and the migrational speed of cells indicate an increased potential for cell invasion under dynamic culture condition^37,41^. For the first time, our study demonstrates that dynamic culture impacts the 3D morphology of spheroids, with spheroids appearing flatter and exhibiting smaller cores compared to static condition. Numerous previous studies have shown that a lower signal intensity inside the spheroid could result from a bigger necrotic core, which is a feature of aged spheroids and is caused by the accumulation of metabolic waste products and inadequate diffusion of oxygen and nutrients to the spheroid’s center^42–44^. Therefore, the continuous flow of oxygen and nutrients in the system may prevent the growth of the necrotic core. Another factor is biophysical forces caused by dynamic culture, which may impact cells. For instance, employing interstitial fluid flow in some systems has revealed that various surface receptors, channels and mechanosensors detect these stimuli, subsequently initiating different signal transduction pathways^45,46^. These findings highlight the importance of dynamic culture in shaping spheroid morphology and its potential implications for subsequent investigations such as drug screening.

Despite variations in experimental designs and cell types employed, it is widely observed that proliferation tends to elevate in response to dynamic culture condition. For instance, the number of MDA-MB-231 cells distributed within a collagen hydrogel increased under dynamic culture condition^37^. The heightened level of the Ki67 marker in MDA-MB-231 and SKBR3 cells under dynamic culture condition also suggests an increase in proliferation^47^. The increased proliferation in PC3 prostate cancer cells seeded in a flow bioreactor without matrix was confirmed through measurement of DNA content^48^. Aligned with other studies, our findings validate the established conclusions within this field, demonstrating through pHH3 staining that the number of proliferative cells is higher in dynamic culture condition. The reason behind the heightened cell proliferation within this system remains unexplored. However, some studies have suggested reasons such as improved oxygen and nutrient diffusion^37^. Additionally, in systems that have modeled interstitial flow, it has been stated that the pressure on embedded cells might drive cell proliferation^49^.

However, there have been conflicting reports regarding apoptosis levels in these systems. MDA-MB-231 and SKBR3 cells distributed within collagen I and exposed to a flow rate of 550 µl/min exhibited upregulation of pro-apoptotic markers such as caspase-3 and caspase-9, alongside significant downregulation of the anti-apoptotic marker Bcl2^47^. Another study demonstrated that a higher flow rate (0.2 ml/min compared to 0.05 ml/min) triggers apoptosis through TGF-β1-mediated signaling in PC3 cells cultured in a flow bioreactor^48^. However, in this bioreactor, cells were directly exposed to the fluid flow without matrix. Unlike HCS-2 cells, Ca Ski and ME-180 cells subjected to dynamic flow induced by a rotary shaker displayed a decrease in the number of cleaved caspase-3-positive cells, indicative of reduced apoptosis^50^. Our study revealed fewer apoptotic cells in HCT-116-GFP spheroids under dynamic culture condition. Collectively, the level of apoptosis in these systems appears to be primarily influenced by factors such as cell line, experimental parameters, and the environment of cells.

Given the evident influence of dynamic culture in increased migration area in this study, investigating genes involved in EMT became imperative. We hypothesized that the expression of key genes in invasion and metastasis might be modified in our system. Considering the significance of genes Snail and MMP9 in EMT, we aimed to understand how alterations in their expression might correlate with the increased migration observed in our study. Snail is a transcription factor with a high expression in colorectal cancer^29,51^. It is also associated with metastasis and poor prognosis. MMP9 is known to promote tumor cell invasion and metastasis through degradation of ECM^52^. Multiple studies also have shown that there is a close connection between MMP9 and Snail expression^53–55^. Our findings revealed a noteworthy pattern in the expression of Snail and MMP9. While upregulation in gene expression was not apparent on day 3, the initial stage of our study, it became prominent on day 7. It has been shown that collagen I in the tumor microenvironment can upregulate the PI3K/AKT signaling pathway through the membrane surface receptor integrin α2β1 in HCT-116 cells. Activation of this signaling pathway induces downstream genes, including Snail, thereby facilitating the invasion and metastasis of colorectal cancer^56^. This mechanism might explain the increased expression of Snail in the embedded HCT-116 spheroids presented in this work. Although upregulation of MMP9 and Snail in the presence of the matrix have been reported in many studies^57–59^, there are limited studies comparing the expression of these genes under static and dynamic culture conditions. Examining the gene expression of MDA-MB-231 cells in a “Quasi-Vivo” flow chamber showed no change in Snail expression, while increased invasion under dynamic culture condition attributed to the upregulation of MMP14^47^. In another study, a non-significant increase in MMP9 expression after 4 days was reported^14^. Differences in gene expression across studies may result from different cell lines and varying conditions, such as direct exposure to shear stress with different levels and durations^48,60–62^. Although different studies have shown that the expression of Snail, MMP9, Slug, Vimentin, N-cadherin, Integrin beta-4 and Catenin beta-1 increases during EMT^2,31,32^, our study demonstrated that only Snail and MMP9 increased under dynamic conditions. These findings could indicate that a complete EMT process did not occur, but rather a partial activation of EMT. Current research suggests that EMT and MET do not occur as clear-cut transitions between purely epithelial and purely mesenchymal states. Instead, they are characterized by multi-state processes, transitioning through one or more intermediate phenotypes, the specifics of which remain debated^5^. Cells in our system may need an extended cultivation period for complete EMT. Nevertheless, further research is necessary to understand the EMT process in dynamic culture condition and to determine which pathways are affected.

All efforts in this research were directed toward the development of a dynamic 3D cell culture system using HCT-116 spheroids as a proof of principle. However, this system holds the potential for further modifications to address existing limitations. For instance, using various types of cells could create a more in vivo-like microenvironment. Given that the co-culturing cancer cells with endothelial cells in microfluidic systems has demonstrated increased cancer cell migration^21^, these cells can be incorporated in the future studies. Continuous monitoring of changes in cell metabolism could be achieved by integrating specific sensors within the system. Moreover, the versatility of this cultivation chamber and system shows potential for applications in tissue culture.

In conclusion, the presented dynamic 3D cell culture system provides an in vivo-like environment by embedding spheroids into a matrix in the presence of fluid flow. Our findings demonstrate that HCT-116 spheroids are significantly influenced by dynamic culture, both morphologically and in terms of gene expression changes. Although our current study focused on the impact of dynamic culture on cell migration behavior, this cultivation chamber hold promise for broader applications. These include exploring the effect of chemotherapy drugs on cancer cells and studying cell–cell or cell-ECM interactions. This system also allows for the parallel use of multiple chambers, enabling the cultivation of more cells and spheroids or the use of various hydrogels simultaneously in a single setup. Consequently, it can be a reliable platform for advancements in the realms of cell biology and tissue engineering.

## Methods and materials

### Dynamic 3D cell culture system and cultivation chamber design and fabrication

In this system, the cultivation chamber is manufactured of different polycarbonate pieces, a glass on the bottom of the well and an aluminum piece for the lower layer. To prevent spheroids from adhering to the bottom of the well, the glass was coated with two layers of poly(2-hydroxyethyl methacrylate) (Merck, USA), each with a concentration of 20 mg/ml. The lid of the cultivation chamber was tightly closed after filling the well with the collagen I matrix and spheroids. A motor was magnetically coupled with a radial pump with adjustable output voltage range up to 9 V. This pump, which is made of polyetheretherketone (PEEK), provides continuous flow in the closed system. The generated flow was measured using a flow sensor SLF3S-1300F (Sensirion, Switzerland) and the flow rate was displayed with Sensirion Viewer software (Sensirion, Switzerland). Setting the voltage to 3 V allows the pump to achieve a flow rate of 1 ml/min in this system. The different parts of the system (pump, flow sensor and 2 cultivation chambers) were connected together using polytetrafluoroethylene (PTFE) tubes (inner Φ = 1.6 mm) (SCP, Germany). 120 µL of collagen I (1.8 mg/ml) with two 4-day-old HCT-116-GFP spheroids were added into the well of the cultivation chamber for dynamic culture. For gelation, the matrix was incubated at room temperature for 30 min. To fill the system, 4 ml medium was injected using a syringe on top of the pump, with a bottle of medium serving as reservoir. Additionally, bubbles could be removed with the syringe and reservoir. For static condition, identical amounts of collagen and spheroids were added into a constructed mold with the same dimension as the well in cultivation chamber. After gelation, the control samples were transferred into 6 well plate with 4 ml medium.

### Cell culture

The human colon cancer cell line HCT-116 was purchased from the American Type Culture Collection (ATCC). As a fluorescent marker GFP was introduced into the cells using the ViraSafe™ Lentiviral Expression System (Cell Biolabs). The cells were cultured in RPMI-1640 medium (Sigma-Aldrich, Germany) supplemented with 10% fetal bovine serum (PAA, Germany), 1% penicillin–streptomycin (P/S) (Gibco, USA) and 2 mM glutamine (Sigma-Aldrich, Germany), at 37 °C in a humidified atmosphere with 5% (v/v) CO_2_ and 95% (v/v) air. Every 3 to 4 days, cells were passaged by trypsinization (Sigma-Aldrich, Germany).

### Spheroid generation

In order to prepare non-adhesive well plates for generation of spheroids, 0.7% (w/v) agarose powder (Lonza, USA) was dissolved into phosphate-buffered saline and then sterilized by autoclaving. 70 µl of agarose solution was added to each well of a 96 well-plate (LABSOLUTE, Germany). After gelation at room temperature, 500 cells/well with 150 µl medium were seeded and then incubated at 37 °C with 5% (v/v) CO_2_ for 4 days in a humidified incubator.

### Matrix preparation

In this study, three matrices were prepared. The collagen I matrix (1.8 mg/ml) was prepared by mixing 1 M NaOH (Merck, Germany), 1 M HEPES buffer (Merck, Germany), 10 × RPMI-1640 and Rat Tail collagen I (MATRIX BioScience, Germany) with a ratio of 0.8:1:2:16. The gelation occurred after incubation at room temperature for 30 min. The Matrigel^®^ matrix (3 mg/ml) was prepared by diluting the Matrigel^®^ (Corning, USA) in RPMI medium then incubated at 37 °C for 30 min. The mixture of Matrigel^®^-collagen l matrix was prepared by the equal volume of Matrigel^®^ and collagen I with the final concentration of 3 mg/ml and 0.9 mg/ml, respectively.

### Imaging

2D bright field images were acquired with OLYMPUS IX81 microscope (Evident Europe, Germany) and the area of spheroids measured by Closed Polygon Tool in CellSens Dimension software (Evident Europe, Germany). To obtain 3D images of spheroids, light sheet fluorescence microscopy was performed. After gluing (BEST KLEBSTOFFE, Germany) collagen gel containing spheroids to the metal holder, they were immersed in 60% 2,2′-Thiodiethanol (Sigma-Aldrich, Germany) solution with refractive index of 1.44 for clearing. The light path in Zeiss light sheet fluorescence microscope Z.1 (Carl Zeiss Microscopy Deutschland, Germany) was set up with the light source laser line 488 nm, the illumination objective 5x/0.1 foc, the detection optic 5x/0.16 foc, the laser blocking filter LBF 405/488/561/640, the emission filter BP 505–545 and the camera (pco.edge 4.2, PCO Tech, USA). Z-stack images were acquired with one side illumination and were rendered with Arivis-version 4DX64 (Carl Zeiss Microscopy Software Center Rostock, Germany). The images for histological staining and TUNEL assay were acquired using Zeiss Axio Imager.Z2 microscope (Carl Zeiss Microscopy Deutschland, Germany) and the images were analyzed by Fiji^63^.

### Extraction of single cells from embedded spheroids

To separate the spheroids from the collagen gel, collagenase treatment was performed. Collagen I gel was incubated in 500 µl of 1.5 mg/ml collagenase (Gibco, USA) at 37 °C for 45 min. Separated spheroids were dissociated to single cells by incubating with 100 µl TrypLE™ Express (Fisher Scientific, Germany) at 37 °C for 10 min. Each step was conducted following a wash with PBS.

### Histology and immunohistochemical (IHC) staining

All samples were fixed with 4% (w/v) formaldehyde (ROTI^®^Histofix, Carl Roth, Germany) at 4 °C overnight. The non-embedded spheroids were implanted into HistoGel (Epredia™ Richard-Allan Scientific™ HistoGel™, Germany) to facilitate their handling during sectioning. Then all the samples underwent dehydration by a series of increasing concentrations of 2-propanol (Carl Roth, Germany). Then they were cleared with ROTI^®^Histol (Carl Roth, Germany) and infiltrated with molten paraffin (ROTI^®^Plast with DMSO, Carl Roth, Germany). After embedding in paraffin wax, sections were prepared with a 5 μm thickness. To deparaffinize samples, they were incubated in ROTI^®^Histol and reducing 2-Propanol and ethanol series. Then samples were stained with Mayer’s hematoxylin (Morphisto, Germany) for 3 min and 1% (w/v) Eosin (Morphisto, Germany) for 5 min. Proliferative cells were identified with pHH3 staining. Boiling sodium citrate buffer (10 mM, pH 6.0) was used as an antigen retrieval solution. Permeabilization was conducted using 0.3% (v/v) Triton-x-100 for 10 min. The primary antibody, anti-phospho-histone H3 (Ser10) (Sigma-Aldrich, Germany), was applied overnight at 4 °C following blocking with 3% (w/v) hydrogen peroxide and 1% (w/v) bovine serum albumin (BSA). The secondary antibody, goat anti-rabbit HRP (Horseradish Peroxidase, Jackson Immuno Research, USA), was applied for at 37 °C. The staining was developed with DAB (3,3′-Diaminobenzidine, Vector Laboratories, USA), and nuclei were stained with hematoxylin.

### TUNEL assay

In order to detect apoptosis, TdT-mediated dUTP nick-end labeling (TUNEL) assay was performed. Following deparaffinization, sections were coated for 15 min at 37 °C with proteinase K working solution (0.6 units/ml) in TE buffer. Biotin and avidin (Abcam, USA) were used as blocking reagents. Samples were covered with TdT storage buffer, TdT reaction buffer, Biotin16-dUTP (Jena Bioscience, Germany) and terminal transferase (TdT) (Roche Diagnostics, Germany) for 2 h at 37 °C. Then samples were incubated with Streptavidin-NL637 (R&D Systems, USA) and Hoechst 33342 for 2 h at 37 °C and 15 min at room temperature, respectively.

### RNA isolation and quantitative real-time PCR

Total RNA was extracted using Bio&SELL RNA-Isolation Mini Kit (Bio&SELL, Germany) according to the manufacturer’s instruction. 500 ng of extracted RNA was reverse-transcribed using SuperScript III (Invitrogen, USA). Then the real time PCR was performed with qPCR Mix EvaGreen kit^®^ (Bio&SELL, Germany). The primers used are Snail forward 5′ CCCCAATCGGAAGCCTAACT 3′, reverse 5′ GCTGGAAGGTAAACTCTGGATTAGA 3′, MMP9 forward 5′ GAACCAATCTCACCGACAGG 3′, reverse 5′ GCCACCCGAGTGTAACCATA 3′, N-cadherin forward 5′ CTGGAACATATGTGATGACC 3′, reverse 5′ TGTAAACATGTTGGGTGAAG 3′, Vimentin forward 5′ GGAAACTAATCTGGATTCACTC 3′, reverse 5′ CATCTCTAGTTTCAACCGTC 3′, Slug forward 5′ CAGTGATTATTTCCCCGTATC 3′, reverse 5′ CCCCAAAGATGAGGAGTATC 3′,Catenin beta-1 forward 5′ CAACT AAACAGGAAGGGATG 3′, reverse 5′ CACAGGTGACCACATTTATATC 3′ and Integrin beta-4 forward 5′ AAGAGGCCCATGTCCATCCC 3′, reverse 5′ TCCATCCGGCCATTCACCAG 3′. The expression level of reference gene *HSP90A* with forward primer 5′ TCTGGGTATCGGAAAGCAAGCC 3′ and reverse primer 5′ GTGCACTTCCTCAGGCATCTTG 3′ was used as a reference gene and fold changes were calculated using 2^−ΔΔCT^ method.

### Statistical analysis

All experiments were performed independently at least in three biological replicates. Statistical analysis was performed using GraphPad Prism (GraphPad Software, USA) version 10.0.0 software with Student’s t-test for single comparisons or one-way ANOVA for multiple comparisons. Results were considered as significantly different when *p < 0.05.

### Supplementary Information

Supplementary Figures.

## Data Availability

The data that support the findings of this study is provided within the manuscript and raw data is available on reasonable request from the corresponding author.

## References

[1] Desai, K., Iqbal, S., Pereira, K. & Thirumaran, R. P-185 Global and regional trends in the incidence and mortality of Colorectal Cancer (CRC): Analysis of data from the GLOBOCAN database and SEER database. *Ann. Oncol.* **34**, S81–S82 (2023). 10.1016/j.annonc.2023.04.241

[2] Vu, T. & Datta, P. K. Regulation of EMT in colorectal cancer: A culprit in metastasis. *Cancers* **9**, 171 (2017). 29258163 10.3390/cancers9120171 PMC5742819

[3] Ribatti, D., Tamma, R. & Annese, T. Epithelial-mesenchymal transition in cancer: A historical overview. *Transl. Oncol.* **13**, 100773 (2020). 32334405 10.1016/j.tranon.2020.100773 PMC7182759

[4] Aggarwal, V., Montoya, C. A., Donnenberg, V. S. & Sant, S. Interplay between tumor microenvironment and partial EMT as the driver of tumor progression. *IScience* **24**, 102113 (2021). 33659878 10.1016/j.isci.2021.102113 PMC7892926

[5] Grigore, A. D., Jolly, M. K., Jia, D., Farach-Carson, M. C. & Levine, H. Tumor budding: The name is EMT. Partial EMT. *J. Clin. Med.* **5**, 51 (2016). 27136592 10.3390/jcm5050051 PMC4882480

[6] Perera, F. P. Environment and cancer: Who are susceptible?. *Science* **278**, 1068–1073 (1997). 9353182 10.1126/science.278.5340.1068

[7] Lee, H. J. *et al.* Fluid shear stress activates YAP1 to promote cancer cell motility. *Nat. Commun.* **8**, 14122 (2017). 28098159 10.1038/ncomms14122 PMC5253685

[8] Sun, M. *et al.* 3D cell culture—Can it be as popular as 2d cell culture?. *Adv. NanoBiomed Res.* **1**, 2000066 (2021). 10.1002/anbr.202000066

[9] Kapałczyńska, M. *et al.* 2D and 3D cell cultures–a comparison of different types of cancer cell cultures. *Arch. Med. Sci.* **14**, 910–919 (2018). 30002710 10.5114/aoms.2016.63743 PMC6040128

[10] Park, S. Y., Hong, H. J. & Lee, H. J. Fabrication of cell spheroids for 3D cell culture and biomedical applications. *BioChip J.* **17**, 24–43 (2023). 10.1007/s13206-022-00086-9

[11] Hu, M., Ling, Z. & Ren, X. Extracellular matrix dynamics: Tracking in biological systems and their implications. *J. Biol. Eng.* **16**, 13. 10.1186/s13036-022-00292-x (2022). 35637526 10.1186/s13036-022-00292-x PMC9153193

[12] Sapir, L., Tzlil, S. Talking over the extracellular matrix: How do cells communicate mechanically?. *Seminars in cell & developmental biology.* **71**, 99–105 (2017). 10.1016/j.semcdb.2017.06.010 28630027

[13] Schmidt, S. & Friedl, P. Interstitial cell migration: Integrin-dependent and alternative adhesion mechanisms. *Cell Tissue Res.* **339**, 83–92 (2010). 19921267 10.1007/s00441-009-0892-9 PMC2784868

[14] Lovecchio, J., Pannella, M., Giardino, L., Calzà, L. & Giordano, E. A dynamic culture platform enhances the efficiency of the 3D HUVEC-based tube formation assay. *Biotechnol. Bioeng.* **117**, 789–797 (2020). 31736057 10.1002/bit.27227

[15] Derda, R. *et al.* supported 3D cell culture for tissue-based bioassays. *Proc. Natl. Acad. Sci.* **106**, 18457–18462 (2009). 19846768 10.1073/pnas.0910666106 PMC2773961

[16] Yasuda, R. *et al.* Cell stress reduction by a novel perfusion-culture system using commercial culture dish. *Appl. Sci.* **10**, 95 (2019). 10.3390/app10010095

[17] Navarro, F. A., Mizuno, S., Huertas, J. C., Glowacki, J. & Orgill, D. P. Perfusion of medium improves growth of human oral neomucosal tissue constructs. *Wound Repair Regen.* **9**, 507–512 (2001). 11896993 10.1046/j.1524-475x.2001.00507.x

[18] Mizuno, S., Allemann, F. & Glowacki, J. Effects of medium perfusion on matrix production by bovine chondrocytes in three-dimensional collagen sponges. *J. Biomed. Mater. Res.* **56**, 368–375 (2001). 11372054 10.1002/1097-4636(20010905)56:3<368::AID-JBM1105>3.0.CO;2-V

[19] Uhl, C. G. & Liu, Y. Microfluidic device for expedited tumor growth towards drug evaluation. *Lab Chip* **19**, 1458–1470 (2019). 30888358 10.1039/C8LC01250D PMC6526058

[20] Lee, H., Park, W., Ryu, H. & Jeon, N. L. A microfluidic platform for quantitative analysis of cancer angiogenesis and intravasation. *Biomicrofluidics*10.1063/1.4894595 (2014). 25332739 10.1063/1.4894595 PMC4189585

[21] Ni, B.-S., Tzao, C. & Huang, J.-H. Plug-and-play in vitro metastasis system toward recapitulating the metastatic cascade. *Sci. Rep.* **9**, 18110 (2019). 31792319 10.1038/s41598-019-54711-z PMC6889311

[22] Mani, V. *et al.* Epithelial-to-mesenchymal transition (EMT) and drug response in dynamic bioengineered lung cancer microenvironment. *Adv. Biosyst.* **3**, 1800223 (2019). 10.1002/adbi.201800223 32627339

[23] Lamouille, S., Xu, J. & Derynck, R. Molecular mechanisms of epithelial–mesenchymal transition. *Nat. Rev. Mol. Cell Biol.* **15**, 178–196 (2014). 24556840 10.1038/nrm3758 PMC4240281

[24] van Midwoud, P. M., Janse, A., Merema, M. T., Groothuis, G. M. & Verpoorte, E. Comparison of biocompatibility and adsorption properties of different plastics for advanced microfluidic cell and tissue culture models. *Anal. Chem.* **84**, 3938–3944 (2012). 22444457 10.1021/ac300771z

[25] Fan, R. *et al.* Circulatory shear flow alters the viability and proliferation of circulating colon cancer cells. *Sci. Rep.* **6**, 27073 (2016). 27255403 10.1038/srep27073 PMC4891768

[26] Aguilar Cosme, J. R., Gagui, D. C., Bryant, H. E. & Claeyssens, F. Morphological response in cancer spheroids for screening photodynamic therapy parameters. *Front. Mol. Biosci.* **8**, 784962 (2021). 34869604 10.3389/fmolb.2021.784962 PMC8637197

[27] Majtnerová, P. & Roušar, T. An overview of apoptosis assays detecting DNA fragmentation. *Mol. Biol. Rep.* **45**, 1469–1478 (2018). 30022463 10.1007/s11033-018-4258-9

[28] Kim, J.-Y. *et al.* The value of phosphohistone H3 as a proliferation marker for evaluating invasive breast cancers: A comparative study with Ki67. *Oncotarget* **8**, 65064 (2017). 29029412 10.18632/oncotarget.17775 PMC5630312

[29] Wang, Y., Shi, J., Chai, K., Ying, X. & Zhou, B. The role of snail in EMT and tumorigenesis. *Curr. Cancer Drug Targets* **13**, 963–972 (2013). 24168186 10.2174/15680096113136660102 PMC4004763

[30] Bloom, A. B. & Zaman, M. H. Influence of the microenvironment on cell fate determination and migration. *Physiol. Genom.* **46**, 309–314 (2014). 10.1152/physiolgenomics.00170.2013 PMC4035623 24619520

[31] Tanabe, S., Aoyagi, K., Yokozaki, H. & Sasaki, H. Regulation of CTNNB1 signaling in gastric cancer and stem cells. *World J. Gastrointest. Oncol.* **8**, 592 (2016). 27574551 10.4251/wjgo.v8.i8.592 PMC4980649

[32] Masugi, Y. *et al.* Upregulation of integrin β4 promotes epithelial–mesenchymal transition and is a novel prognostic marker in pancreatic ductal adenocarcinoma. *Lab. Investig.* **95**, 308–319 (2015). 25599535 10.1038/labinvest.2014.166

[33] Perestrelo, A. R., Águas, A. C., Rainer, A. & Forte, G. Microfluidic organ/body-on-a-chip devices at the convergence of biology and microengineering. *Sensors* **15**, 31142–31170 (2015). 26690442 10.3390/s151229848 PMC4721768

[34] Freires, I. A., Sardi, J. C. O., de Castro, R. D. & Rosalen, P. L. Alternative animal and non-animal models for drug discovery and development: Bonus or burden?. *Pharm. Res.* **34**, 681–686. 10.1007/s11095-016-2069-z (2017). 27858217 10.1007/s11095-016-2069-z

[35] Guzman, A., Ziperstein, M. J. & Kaufman, L. J. The effect of fibrillar matrix architecture on tumor cell invasion of physically challenging environments. *Biomaterials* **35**, 6954–6963 (2014). 24835043 10.1016/j.biomaterials.2014.04.086

[36] Medici, D. & Nawshad, A. Type I collagen promotes epithelial–mesenchymal transition through ILK-dependent activation of NF-κB and LEF-1. *Matrix Biol.* **29**, 161–165 (2010). 20018240 10.1016/j.matbio.2009.12.003 PMC2849845

[37] Pasini, A. *et al.* Perfusion flow enhances viability and migratory phenotype in 3D-cultured breast cancer cells. *Ann. Biomed. Eng.* **49**, 2103–2113. 10.1007/s10439-021-02727-w (2021). 33543395 10.1007/s10439-021-02727-w PMC8455496

[38] Osawa, T., Wang, W., Dai, J. & Keller, E. T. Macrofluidic recirculating model of skeletal metastasis. *Sci. Rep.* **9**, 14979 (2019). 31628348 10.1038/s41598-019-50577-3 PMC6802200

[39] Bravo-Cordero, J. J., Hodgson, L. & Condeelis, J. Directed cell invasion and migration during metastasis. *Curr. Opin. Cell Biol.* **24**, 277–283 (2012). 22209238 10.1016/j.ceb.2011.12.004 PMC3320684

[40] Palazzolo, G. *et al.* Modulating the distant spreading of patient-derived colorectal cancer cells via aspirin and metformin. *Transl. Oncol.* **13**, 100760 (2020). 32247264 10.1016/j.tranon.2020.100760 PMC7118176

[41] Haessler, U., Teo, J. C., Foretay, D., Renaud, P. & Swartz, M. A. Migration dynamics of breast cancer cells in a tunable 3D interstitial flow chamber. *Integrat. Biol.* **4**, 401–409 (2012). 10.1039/c1ib00128k 22143066

[42] Han, S. J., Kwon, S. & Kim, K. S. Challenges of applying multicellular tumor spheroids in preclinical phase. *Cancer Cell Int.* **21**, 1–19 (2021). 33663530 10.1186/s12935-021-01853-8 PMC7934264

[43] Kopanska, K. S., Alcheikh, Y., Staneva, R., Vignjevic, D. & Betz, T. Tensile forces originating from cancer spheroids facilitate tumor invasion. *PLoS One* **11**, e0156442 (2016). 27271249 10.1371/journal.pone.0156442 PMC4896628

[44] Štampar, M., Breznik, B., Filipič, M. & Žegura, B. Characterization of in vitro 3D cell model developed from human hepatocellular carcinoma (HepG2) Cell Line. *Cells* **9**, 2557 (2020). 33260628 10.3390/cells9122557 PMC7759933

[45] Shieh, A. C. & Swartz, M. A. Regulation of tumor invasion by interstitial fluid flow. *Phys. Biol.* **8**, 015012 (2011). 21301060 10.1088/1478-3975/8/1/015012

[46] Kim, O.-H., Jeon, T. J., Shin, Y. K. & Lee, H. J. Role of extrinsic physical cues in cancer progression. *BMB Rep.* **56**, 287 (2023). 37037673 10.5483/BMBRep.2023-0031 PMC10230013

[47] Azimi, T., Loizidou, M. & Dwek, M. V. Cancer cells grown in 3D under fluid flow exhibit an aggressive phenotype and reduced responsiveness to the anti-cancer treatment doxorubicin. *Sci. Rep.* **10**, 12020 (2020). 32694700 10.1038/s41598-020-68999-9 PMC7374750

[48] Jasuja, H., Jaswandkar, S. V., Katti, D. R. & Katti, K. S. Interstitial fluid flow contributes to prostate cancer invasion and migration to bone; study conducted using a novel horizontal flow bioreactor. *Biofabrication* **15**, 025017 (2023). 10.1088/1758-5090/acc09a PMC10020972 36863017

[49] Yu, T. *et al.* High interstitial fluid pressure promotes tumor cell proliferation and invasion in oral squamous cell carcinoma. *Int. J. Mol. Med.* **32**, 1093–1100 (2013). 24043259 10.3892/ijmm.2013.1496

[50] Hanashima, K. *et al.* Tissue-specific physical and biological microenvironments modulate the behavior of cervical squamous cell carcinoma. *Acta Histochemica et Cytochemica* **54**, 155–165 (2021). 34764524 10.1267/ahc.21-00038 PMC8569132

[51] Kaufhold, S. & Bonavida, B. Central role of Snail1 in the regulation of EMT and resistance in cancer: A target for therapeutic intervention. *J. Exp. Clin. Cancer Res.* **33**, 1–19 (2014). 25084828 10.1186/s13046-014-0062-0 PMC4237825

[52] Winkler, J., Abisoye-Ogunniyan, A., Metcalf, K. J. & Werb, Z. Concepts of extracellular matrix remodelling in tumour progression and metastasis. *Nat. Commun.* **11**, 5120. 10.1038/s41467-020-18794-x (2020). 33037194 10.1038/s41467-020-18794-x PMC7547708

[53] Abdulkhalek, S. *et al.* Transcriptional factor snail controls tumor neovascularization, growth and metastasis in mouse model of human ovarian carcinoma. *Clin. Transl. Med.* **3**, 1–16 (2014). 26932374 10.1186/s40169-014-0028-z PMC4884043

[54] Lin, C. Y. *et al.* Matrix metalloproteinase-9 cooperates with transcription factor Snail to induce epithelial–mesenchymal transition. *Cancer Sci.* **102**, 815–827 (2011). 21219539 10.1111/j.1349-7006.2011.01861.x

[55] Rho, S. B., Byun, H.-J., Kim, B.-R. & Lee, C. H. Snail promotes cancer cell proliferation via its interaction with the BIRC3. *Biomol. Ther.* **30**, 380 (2022). 10.4062/biomolther.2022.063 PMC9252879 35711139

[56] Wu, X., Cai, J., Zuo, Z. & Li, J. Collagen facilitates the colorectal cancer stemness and metastasis through an integrin/PI3K/AKT/Snail signaling pathway. *Biomed. Pharmacother.* **114**, 108708 (2019). 30913493 10.1016/j.biopha.2019.108708

[57] Miserocchi, G. *et al.* Three-dimensional collagen-based scaffold model to study the microenvironment and drug-resistance mechanisms of oropharyngeal squamous cell carcinomas. *Cancer Biol. Med.* **18**, 502 (2021). 33772505 10.20892/j.issn.2095-3941.2020.0482 PMC8185858

[58] Chen, L., Ma, H., Li, K., Song, X. & Zeng, X. Liver extracellular matrix hydrogel-based three-dimensional culture system of HepG2 cells to enhance cancer stem cell properties. *Mater. Sci. Eng. C* **126**, 112119 (2021). 10.1016/j.msec.2021.112119 34082936

[59] Chen, L. *et al.* The enhancement of cancer stem cell properties of MCF-7 cells in 3D collagen scaffolds for modeling of cancer and anti-cancer drugs. *Biomaterials* **33**, 1437–1444 (2012). 22078807 10.1016/j.biomaterials.2011.10.056

[60] Mahmoud, M. M. *et al.* Shear stress induces endothelial-to-mesenchymal transition via the transcription factor Snail. *Sci. Rep.* **7**, 3375 (2017). 28611395 10.1038/s41598-017-03532-z PMC5469771

[61] Maggiorani, D. *et al.* Shear stress-induced alteration of epithelial organization in human renal tubular cells. *PLoS One* **10**, e0131416 (2015). 26146837 10.1371/journal.pone.0131416 PMC4493045

[62] Liu, S. *et al.* Fluid shear stress induces epithelial-mesenchymal transition (EMT) in Hep-2 cells. *Oncotarget* **7**, 32876 (2016). 27096955 10.18632/oncotarget.8765 PMC5078059

[63] Schindelin, J. *et al.* Fiji: An open-source platform for biological-image analysis. *Nat. Methods* **9**, 676–682. 10.1038/nmeth.2019 (2012). 22743772 10.1038/nmeth.2019 PMC3855844

