# Supplementary material for: Dynamic cell culture modulates colon cancer cell migration in a novel 3D cell culture system
Source: Sci Rep. 2024 Aug 14;14:18851. doi: 10.1038/s41598-024-69261-2 (PMC11324956; doi:10.1038/s41598-024-69261-2)
Supplement: Supplementary file 1 — Supplementary Figures. [file 41598_2024_69261_MOESM1_ESM.pdf]

Supplementary Information

Dynamic cell culture modulates colon cancer cell migration in a novel 3D cell culture system

M. Mohamadian Namaqi*, F. Moll, S. Wiedemeier, A. Grodrian, K. Lemke

Department of Bioprocess Engineering, Institute for Bioprocessing and Analytical Measurement Techniques e.V. (iba), Heilbad Heiligenstadt, Germany

*Corresponding author: Masoumeh.Namaqi@iba-heiligenstadt.de

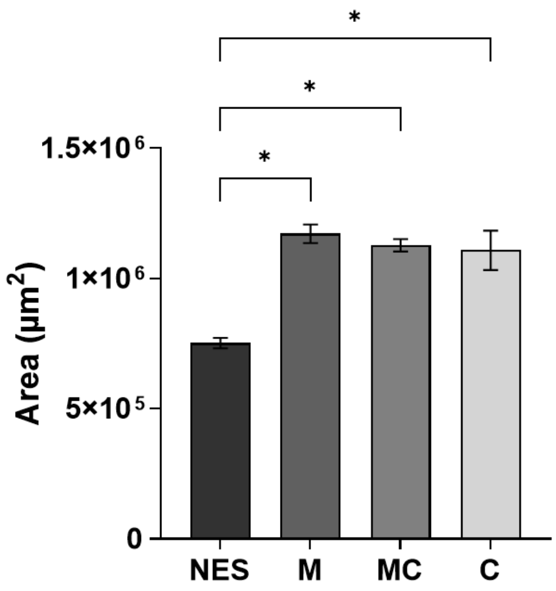

Figure S1. Area of HCT-116-GFP spheroids in different matrices. NES: non-embedded spheroids, M: Matrigel®, C: collagen I and MC: mixture of Matrigel® and collagen I on day 7, n = 3. Data are shown as mean ± SD (* = $P < 0.05$) .

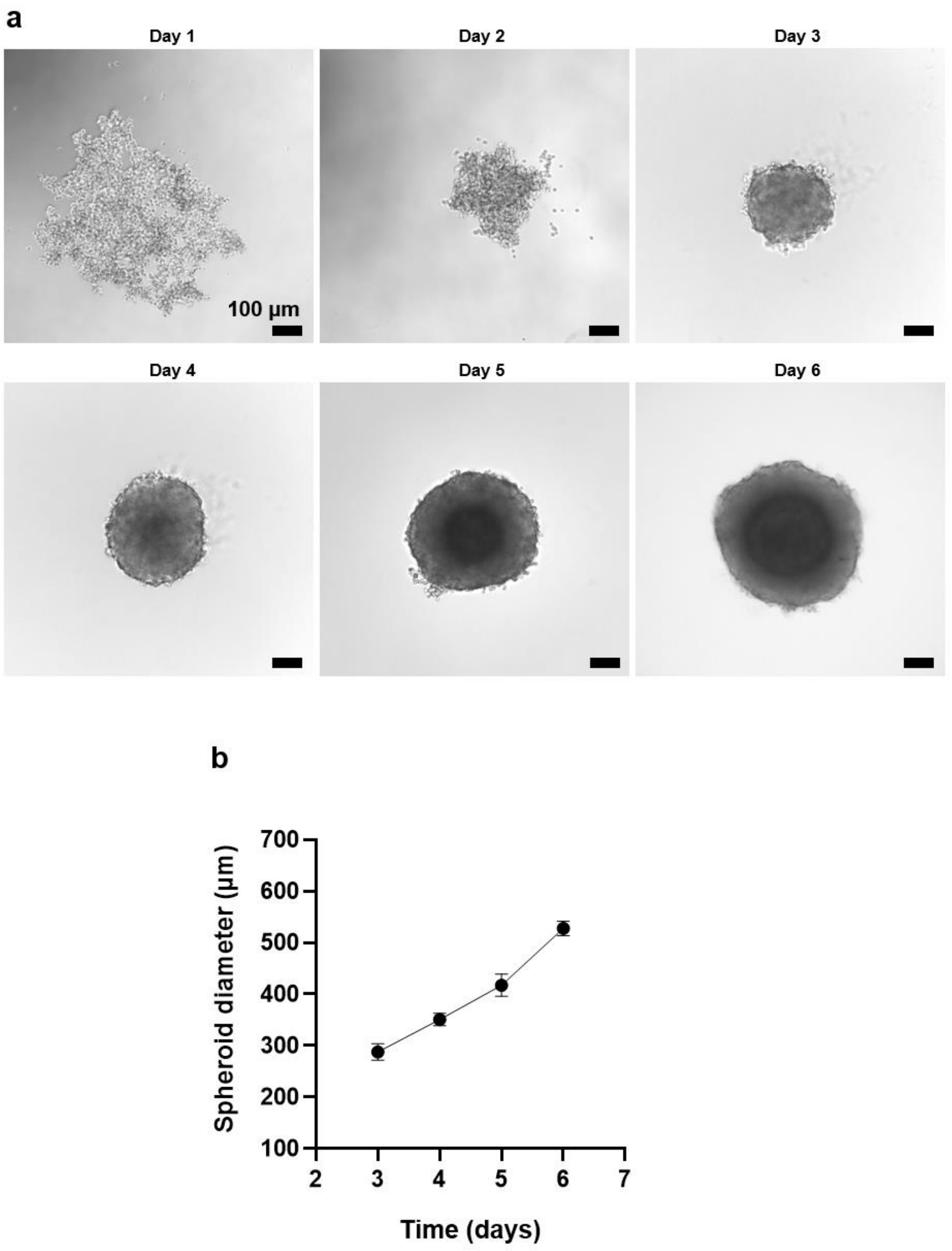

Figure S2. Generation of HCT-116-GFP spheroid in 96 well-plate. (a) Representative images of HCT-116-GFP spheroids during 6 days. The spheroids were fully developed and large enough by day 4, thus they were chosen for transfer and embedding in collagen gel. Scale bar: 100 µm. (b) Diameter of spheroids in different days. n = 3. Data are shown as mean ± SD.

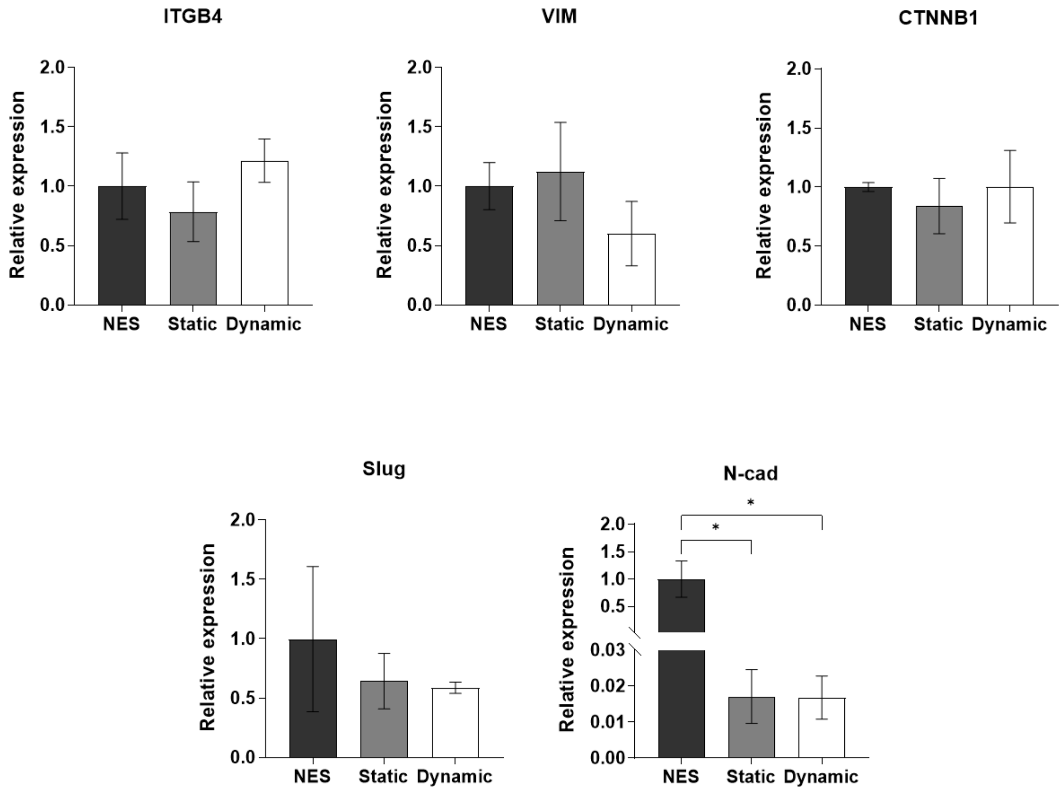

Figure S3. Gene expression analysis of HCT-116 spheroids in static and dynamic culture conditions on day 7. ITGB4: Integrin beta-4, VIM: Vimentin, CTNNB1: Catenin beta-1, Slug: Slug, N-cad: N-cadherin. Gene expression was analyzed by qPCR using HSP90AB as reference gene (2^-ΔΔCT method). Data are shown as mean ± SD. n = 3 (* p = < 0.05).
